# A Predictive Nomogram for Lymph Node Metastasis in Supraglottic Laryngeal Squamous Cell Carcinoma

**DOI:** 10.3389/fonc.2022.786207

**Published:** 2022-03-02

**Authors:** Lulu Song, Yu Heng, Chi-Yao Hsueh, Huiying Huang, Lei Tao, Liang Zhou, Ming Zhang

**Affiliations:** Department of Otolaryngology, Shanghai Key Clinical Disciplines of Otorhinolaryngology, Eye & ENT Hospital, Fudan University, Shanghai, China

**Keywords:** supraglottic squamous cell laryngeal cancer, lymph node metastasis, nomogram, diagnosis, C-index

## Abstract

**Purpose:**

Lymph node metastasis (LNM) has a negative impact on the survival of patients with laryngeal squamous cell carcinoma (LSCC). Supraglottic LSCC is the most common cause of cervical lymph node metastases due to the extensive submucosal lymphatic plexus. The accurate evaluation of LNM before surgery can inform improved decisions in the clinic. In this study, we aimed to construct a nomogram to predict LNM in primary supraglottic LSCC patients.

**Methods:**

The data from 314 patients with clinico-pathological confirmed supraglottic LSCC who underwent partial or total laryngectomy in our department from 2016 to 2020 were retrospectively analyzed (243 cases in the training set and 71 cases in the validation set). A multivariate logistic regression model was used to screen out independent risk factors and a nomogram was established. The accuracy and discrimination ability of the nomogram was evaluated using a consistency index and calibration curves.

**Results:**

Tumor size, tumor differentiation degree and LMR (lymphocyte-monocyte ratio) were selected to construct the nomogram. The C-index was 0.731 in the training set and 0.707 in the validation set. The calibration curves of the training and validation group both exhibited close agreement between the predicted and the actual presence of LNM.

**Conclusions:**

A nomogram was established based on routinely measured pretreatment variables and the predicted results improved the management of patients with LNM.

## Introduction

Laryngeal cancer (LC) is one of the most common tumors of the respiratory tract (1). LC can be anatomically subdivided into glottic, supraglottic, and subglottic cancer based on its primary site. 60-70% of cases originate from the glottis and approximately 35% of cases originate from the supraglottic site (2). The supraglottic area is characterized by a rich lymphatic network resulting in a high potential for the development of regional metastases (3). The involvement of metastatic cervical lymph nodes has been shown to negatively impact survival (4). In clinical practice, positive lymph nodes may be palpable or can be detected by ultrasonography, computed tomography (CT), or magnetic resonance imaging (MRI). However, false positive results are frequently caused by inflammatory conditions whilst false negatives can be due to the small size of metastatic lymph nodes or cystic changes (5).

Several studies have identified indicators that may be independent factors for LNM such as the tumor depth, the degree of tumor differentiation, T-stage, thyroid cartilage invasion, and extra laryngeal extension. Traditional methods for determining the factors related to LNM are largely qualitative and there remains a need to develop quantitative measures to assess the factors associated with the risk of LNM (6, 7). The accurate preoperative evaluation of LNM risk may guide the use of optimized treatment strategies in patients with supraglottic LSCC and provide important prognostic information. In this study, we retrospectively analyzed data from 314 patients with supraglottic LSCC admitted to hospital between 2016 and 2020. These data were used to develop a nomogram prediction model for LNM in supraglottic LSCC patients.

## Methods

### Patient Cohort

This study retrospectively collected 314 clinical cases of newly diagnosed primary supraglottic LSCC confirmed by postoperative pathology in the Eye, Ear, Nose, and Throat Hospital of Fudan University. We defined the training and the validation groups by time in this study. The training group consisted of 243 patients who were admitted between January 2016 and December 2018 and the validation group consisted of 71 patients who were hospitalized between January 2019 and December 2020. The training group was used to establish the model, and the validation group was used to verify the performance of the model. The inclusion criteria for the study were as follows: (1) supraglottic laryngeal squamous cancer confirmed by postoperative pathology; (2) no preoperative chemotherapy or radiotherapy; (3) complete clinical and pathological data; (3) no history of other cancers; (4) no distant metastasis.

This study was approved by the Institutional Ethics Committee of the Eye and ENT Hospital of Fudan University.

### Surgical Treatments and Data Collection

Clinical and pathological data including the demographic data, blood test report, tumor size, clinical tumor stages, and differentiation grades were collected. The following pretreatment hematological parameters were collected within 4 weeks before initial treatment: neutrophil, lymphocyte, monocyte and platelet counts. The platelet-lymphocyte (PLR), neutrophil-lymphocyte (NLR) and lymphocyte-monocyte (LMR) ratios were calculated by dividing the absolute values of the corresponding hematological parameters. The degree of tumor differentiation was obtained by pre-operative biopsy. The postoperative pathology reports were screened to confirm whether the patients met the criteria for inclusion. Tumor size was defined as the maximum diameter of the primary tumor based on computed tomography measurements. Staging was performed according to AJCC 8th edition guidelines.

**Figure 1:**
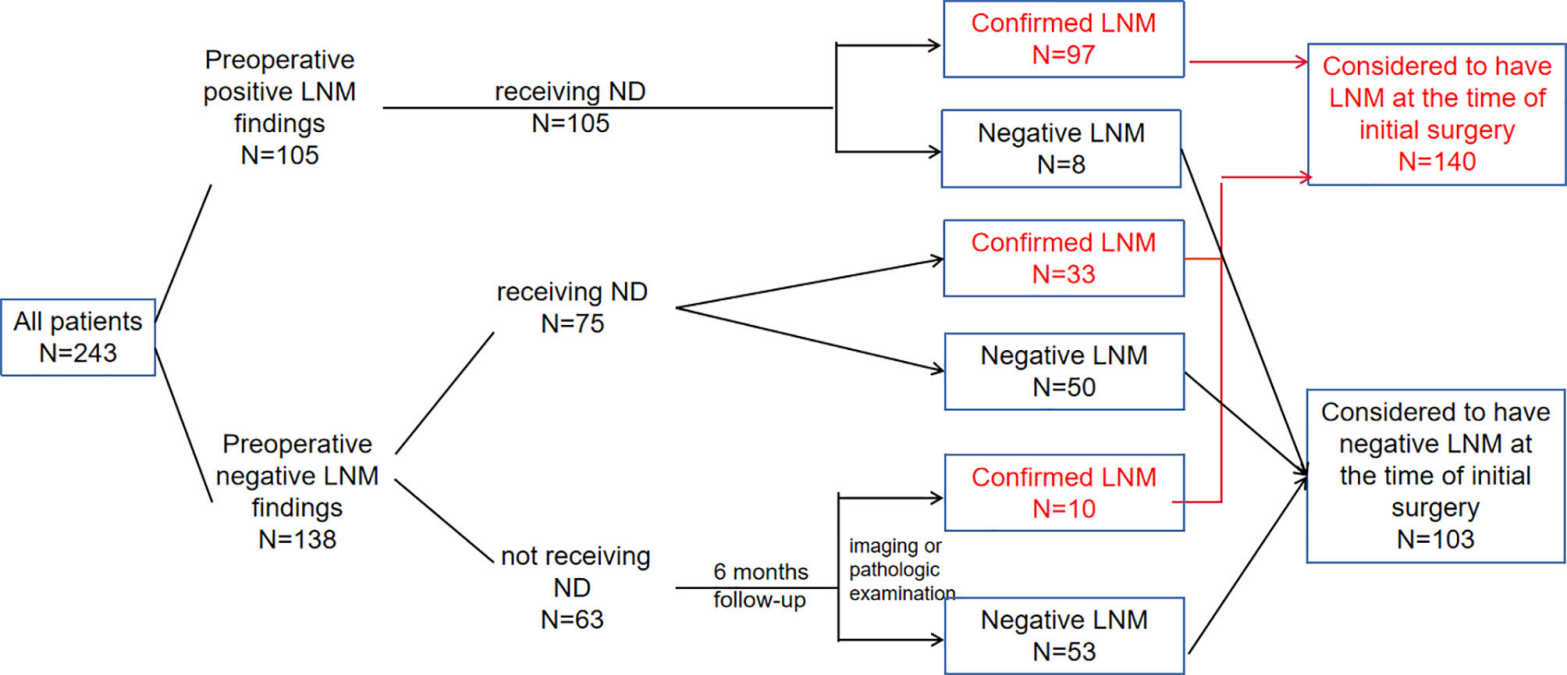
Treatment management, postoperative pathology and follow-up information for patients in the training group.

The primary tumor resection was conducted for all patients in our study, while neck dissection was performed therapeutically or prophylactically in 180 patients. In patients receiving neck dissection, lymph node status (no metastasis, N0, or lymph node metastasis, N+) was verified based on the final pathological assessments. In 63 patients who didn’t receive neck dissection, if positive LNM was found by postoperative follow-up six months after initial operation, they were regarded as having occult lymph node involvement at the time of initial surgery, and thus be classified as LNM group (**Figure 1**).

### Variable Analysis

The variables analyzed in this study included the following clinicopathologic data: sex, age, drinking history, smoking history, tumor size, differentiation, PLR, NLR and LMR. The optimal cut-off values were calculated according to the receiver operating characteristic (ROC) curve. Based on the cut-off values, the continuous variables were transformed into categorical variables.

### Statistical Analysis

A Chi-square test was used to compare the categorical variables. Multivariable logistic regression analysis was performed with the following clinical and pathological candidate predictors: age, gender, tumor size, tumor differentiation degree, LMR, NLR and PLR, which were applied to develop a diagnostic model for LNM using the primary cohort. A bi-direction stepwise selection process with the Akaike information criterion as the stopping rule was performed. The nomogram was formulated based on the results. To evaluate the discrimination of our predictive model, the concordance index (C-index) and receiver operating characteristic (ROC) curve were constructed, and a calibration curve was used to assess the consensus degree of our models. In this study, SPSS 26.0 and R software (version 3.6.1, www.rproject.org) were used in statistical analyses.

## Results

### Patients Characteristics

The characteristics of the training and validation groups are shown in **Table 1**. Between 2016 and 2018, 243 patients served as the training group to create the predictive model. The other 71 patients admitted between 2019 and 2020 served as external validation group for verifying the model. The mean age of all patients enrolled was 64 years, 63 years for training and 66 years for validation group. In all patients, the cervical metastasis rates are 45.9% with early-stage (pT1/2) and 64.2% in patients with pT3/4 tumors. There were no significant differences between the two cohorts in LNM prevalence (P = 0.493). LNM positivity was 57.6% in the primary cohort and 52.1% in the validation cohort. The cut-off values (PLR = 129.41, NLR = 2.76, LMR= 3.12, tumor size = 2.7) were calculated according to the receiver operating characteristic (ROC) curve. Based on the cut-off values, the continuous variables were transformed into categorical variables. In the training group, LNM was associated with the following clinicopathological parameters: tumor differentiation degree (P<0.001), LMR (P = 0.023), clinical T stage (P = 0.031), NLR (P=0.05) and tumor size (P<0.001) (**Table 2**).

**Table 1:** Clinicopathological characteristics of all patients.

Variables	Total (314)	Training (243)	Validation (71)	p
**HBP**				< 0.01
**NO**	221	186	35	
**Yes**	93	57	36	
**DM**				0.07
**NO**	285	225	60	
**Yes**	29	18	11	
**Smoking**				0.53
**NO**	59	48	11	
**Yes**	255	195	60	
**Drinking**				1.00
**NO**	131	101	30	
**Yes**	183	142	41	
**Sex**				0.05
**Female**	14	14	0	
**Male**	300	229	71	
**cT stage**				0.05
**1**	11	8	3	
**2**	124	98	26	
**3**	154	113	41	
**4**	25	24	1	
**Age**				0.10
**<65**	171	139	32	
**≥65**	143	104	39	
**Grading**				0.16
**moderate to high**	50	43	7	
**moderate**	264	200	64	
**TS**				0.46
**<2.7**	123	92	31	
**≥2.7**	191	151	40	
**NLR**				1.00
**<2.76**	193	149	44	
**≥2.76**	121	94	27	
**LMR**				0.29
**<3.12**	118	87	31	
**≥3.12**	196	156	40	
**PLR**				1.00
<129.41	158	122	36	
≥129.41	156	121	35	

HBP, High blood pressure; DM, Diabetes mellitus; TS, tumor size; PLR, Platelet/lymphocyte; NLR, Neutrophil/lymphocyte; LMR, Lymphocyte.

**Table 2:** Relationship between lymph node metastasis and clinicopathologic variables in training set.

Variables	Total n = 243	LNM(-) n = 103	LNM(+) n = 140	P-value
HBP				0.102
NO	186	73	113	
Yes	57	30	27	
DM				1
NO	225	95	130	
Yes	18	8	10	
Smoking				0.482
NO	48	23	25	
Yes	195	80	115	
Drinking				0.479
NO	101	46	55	
Yes	142	57	85	
Sex				0.153
Female	14	9	5	
Male	229	94	135	
CT stage				0.031
1	8	7	1	
2	98	45	53	
3	113	41	72	
4	24	10	14	
Age				0.143
<65	139	65	74	
≥65	104	38	66	
Grading				<0.001
moderate to high	43	30	13	
moderate	200	73	127	
TS				< 0.001
<2.7	92	54	38	
≥2.7	151	49	102	
NLR				0.05
<2.76	149	71	78	
≥2.76	94	32	62	
LMR				0.023
<3.12	87	28	59	
≥3.12	156	75	81	
PLR				0.956
<129.41	122	51	71	
≥129.41	121	52	69	

HBP, High bloodpressure; DM, Diabetes mellitus; TS, tumor size; PLR, Platelet/lymphocyte; NLR, Neutrophil/lymphocyte; LMR, Lymphocyte/ monocyte; CT stage, clinical Tumor Stage.

### Surgical Treatments and Follow-up Information

All patients received partial or total laryngectomy and neck dissection was performed in patients with positive or highly suspicious LNM. In the training group, neck dissection was performed on 180 (74.1%) patients, 105 of which had clinically detectable LNM. 33 of the 75 patients who were preoperatively negative but highly suspicious LNM were found to have LNM on postoperative pathology. In patients that did not receive neck dissection, 10 out of 63 were diagnosed with LNM by imaging tests or pathologic examination during the postoperative 6 months follow-up. In total, 140 (57.6%) patients were regarded as having LNM at the time of initial treatment (**Figure 1**).

### Risk Factors for LNM and Construction of the Nomogram

**Figure 2:**
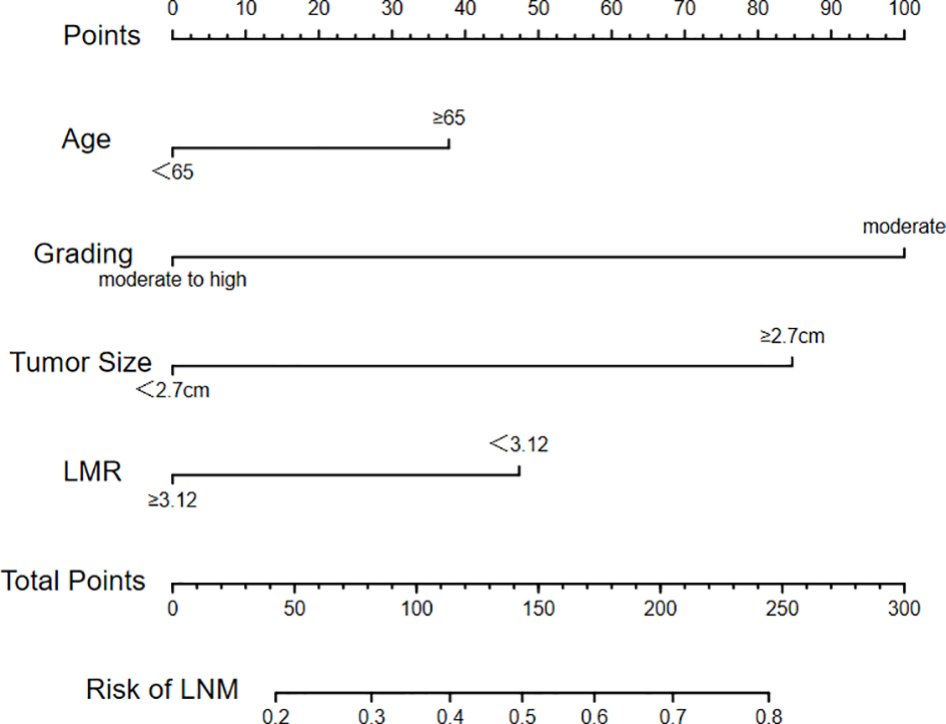
Nomogram constructed according to selected variables.

Multivariable logistic regression analysis was performed with the following clinical and pathological candidate predictors: age, gender, tumor size, tumor differentiation degree, LMR, NLR and PLR. The results indicated that tumor differentiation degree (OR=3.752, P=0.001), tumor size (OR=3.103, P<0.001) were associated with LNM. Patients older than 65 years were more likely to have LNM with an odds ratio of 1.692. Patients with a LMR greater than 3.12 were less likely to develop LNM with an odds ratio of 0.562 (**Table 3**). The multivariate logistic model was used to develop a diagnostic model for LNM using the training cohort, and a bi-direction stepwise selection process was performed to select variables with the Akaike information criterion as the stopping rule. Finally, tumor differentiation degree, age, LMR and tumor size were selected to establish the nomogram to predict the risk of LNM in patients with newly diagnosed primary supraglottic LSCC cancer (**Figure 2**).

**Table 3:** Multivatiate logistic regression analysis for predicting lymph node metastasis.

Variables		95%C1	P
Sex	Female	–	
	Male	1.360 (0.426-4.811)	0.612
Age	<65	–	
	≥65	1.692 (0.958-3.028)	0.072
Grading	moderate to high	–	
	moderate	3.752 (1.790-8.246)	0.001
Tumor size	<2.7	–	
	≥2.7	3.103 (1.750-5.594)	< 0.001
PLR	<140.84	–	
	≥140.84	0.668 (0.347-1.267)	0.22
NLR	<2.76	–	
	≥2.76	1.572 (0.786-3.180)	0.203
LMR	<3.12	–	
	≥3.12	0.562 (0.285-1.093)	0.092

PLR, Platelet/lymphocyte; NLR, NeutrophiVlymphocyte; LMR, Lymphocyte/ monocyte.

### Evaluation and Validation of the Nomogram

**Figure 3:**
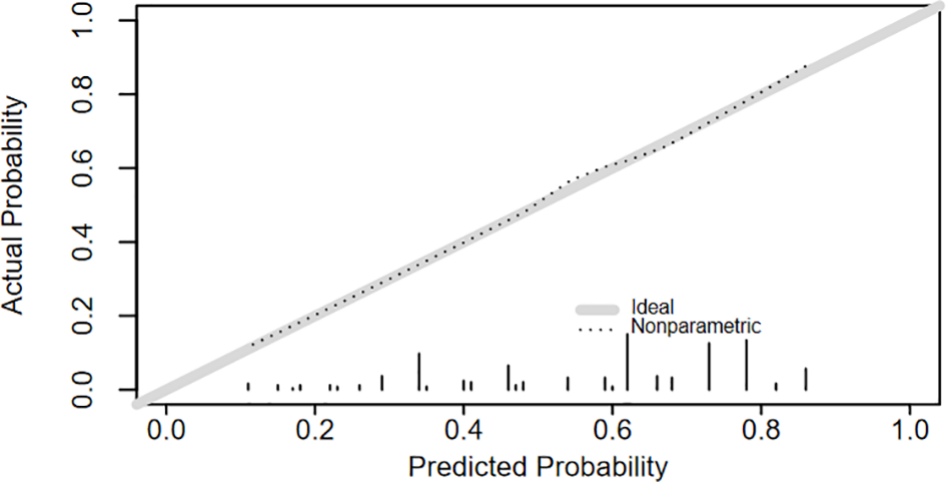
The calibration curve of the nomogram for predicting LNM in training group.

**Figure 4:**
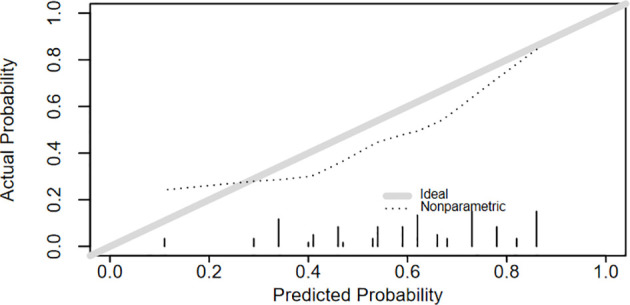
The calibration curve of the nomogram for predicting LNM in test group.

A logistic regression model was used to develop a multivariate model to predict the LNM of the patients. Each variable corresponded to a specific point by drawing a straight line upwards to the point axis. All of the points were added to obtain the total. Finally, the risk value corresponding to the total score was determined. For example, the total score of moderate differentiation with TS≥2.7 cm and age≥65 plus LMR≥3.12 in patients with supraglottic LSCC was 37 + 84+100+0 = 221. Then, the corresponding risk for LNM was 73% (**Figure 2**). The bootstrap method was used to evaluate the precision of the nomogram internally and externally. The C-index was 0.731 in the training set and 0.707 in the validation set. The calibration curves of the training and validation group are displayed in **Figures 3**, **4**. Both exhibited satisfying accordance between the predicted and the actual presence of LNM.

## Discussion

Supraglottic LSCC is commonly associated with cervical LNM due to the extensive submucosal lymphatic plexus (8). According to a previous study, high cervical metastasis rates are common across all stages of supraglottic laryngeal cancer ranging from 55% in patients with early-stage (pT1/2) to 67% in patients with pT3/4 tumors (9). In our study, the rate of LNM rates were 45.9% and 64.2%, respectively for these stages of the disease. LNM has been shown to correlate with a high risk of distant metastases and the number of metastatic nodes is a predominant independent factor of mortality (10). Allen et al. found that the risk of mortality escalated continuously with an increasing number of metastatic nodes. Also, the hazard per node (hazard ratio [HR], 1.19; 95% CI, 1.16-1.23; P < 0.001) was most pronounced when up to 5 positive lymph nodes were detected (11). These data suggest that cervical lymph node management is vital and identifying the objective determinants for LNM could lead to the development of improved individualized therapy decisions.

The current clinically established methods for detecting LNM have several limitations. As for the physical examination, the sensitivity and specificity of findings are unsatisfactorily low with false-negative rates as high as 15-25% and similar false-positive rates (12). In contrast, the detection of LNM by radiological imaging is more accurate compared to clinical examination. Commonly, CT is used for the staging of lymph nodes in the neck. The criteria for assessing nodal metastases include nodal size, shape, presence of central necrosis, and grouping of nodes in an expected draining nodal station (5, 13). However, imaging assessment of LNM in the head and neck can be challenging for the radiologist as there are multiple cervical levels to review and various criteria have been proposed (14). False-positive results can be caused by inflammatory conditions and false-negative results due to small size and cystic change of metastatic lymph nodes (5). As for the comparison of CT and MR imaging, it showed no significant difference between the two imaging tests for either sensitivity (P = 0.1317) or specificity (P = 0.3173) (15). PET/CT can be used to achieve a 21% increase in the diagnosis of nodal metastasis compared with conventional images yet it has limited cost-effectiveness (16). Accordingly, a practical and comprehensive prediction model that integrates multiple indicators could facilitate the accurate assessment of LNM in patients with supraglottic LSCC.

In this study, the four variables obtained before surgery (age, tumor size, tumor differentiation degree and LMR) were selected to construct the nomogram. The continuous variables were transformed into categorical variables with the optimal cut-off values (PLR = 129.41, NLR = 2.76, LMR= 3.12, tumor size = 2.7) calculated according to the receiver operating characteristic (ROC) curve. Several previous studies have shown that patients with poorly differentiated and larger primary tumors have a higher incidence of lymph node involvement (17, 18). Consistent with previous studies, two primary related factors including a maximum tumor diameter ≥ 2.7 cm and poorly differentiated tumors were shown to be independent risk factors for LNM in supraglottic LSCC patients. Of the hematological parameters assessed in this study, our data showed that patients with a LMR lower than 3.12 were more likely to develop LNM. This may be due to the ability of monocytes to secrete various proinflammatory cytokines that promote tumorigenesis, angiogenesis and distant metastasis and low lymphocyte levels are associated with poor tumor control (19, 20).

The National Comprehensive Cancer Network (NCCN) Clinical Practice Guidelines specify that patients with supraglottic lesions should have neck treatment even in N0 cases. However, Sessions et al. conducted a retrospective study of 653 patients with supraglottic laryngeal squamous cell cancer and found that patients with N0 disease may be safely observed with no loss of survival advantage (21). Also, Ömer et al. found a very low incidence of LNM in T1-T2 stage and well-differentiated tumors. These data suggested that a watchful waiting strategy can be used in T1-T2 and selected T3 cases with well-differentiated tumors (6).

Elective neck dissection is widely accepted as the standard surgical treatment for clinically node-negative patients (22). However, neck dissection may result in complications such as recurrent laryngeal nerve palsy (while clearing central compartment nodes in partial laryngectomy), hematoma, chyle leakage, and spinal accessory nerve dysfunction (23). This approach is a form of overtreatment in patients that have no lymph node involvement. Based on our nomogram, the individual risk of LNM can be determined and doctors can identify patients with a high risk of LNM. The model can be used to avoid overtreatment and reduce the risk of dissection–related complications. Also, our nomogram can directly inform the lymph node dissection strategy for those with a high risk of occult LNM.

To the best of our knowledge, this is the first study to develop a nomogram to predict LNM in supraglottic LSCC, which can be used to predict the individual risk of LNM and to identify patients with a high LNM risk. It can be useful in evaluating the optimized treatment strategies and provide important prognostic information. However, our study had several limitations. Our study was performed as a retrospective study and may have had inherent section bias. Also, all of the enrolled patients were from a single institution which may be a source or bias. Multicentre studies are required to validate our model.

## Conclusion

Based on tumor differentiation degree, age, LMR and tumor size, a nomogram model was established to predict the incidence of LNM in patients with supraglottic LSCC. This model has potential value in predicting the LNM risk. However, further multicentre studies with larger samples are needed to validate these findings.

## Data Availability Statement

The raw data supporting the conclusions of this article will be made available by the authors, without undue reservation.

## Ethics Statement

The studies involving human participants were reviewed and approved by the Institutional Ethics Committee of the Eye and ENT Hospital of Fudan University. Written informed consent to participate in this study was provided by the participants’ legal guardian/next of kin.

## Author Contributions

The data was collected and analyzed by LS. YH, C-YH, and HH helped design the project. The manuscript was written by LS, and revised by MZ, LT, and LZ. All authors contributed to the article and approved the submission.

## Conflict of Interest

The authors declare that the research was conducted in the absence of any commercial or financial relationships that could be construed as a potential conflict of interest.

## Publisher’s Note

All claims expressed in this article are solely those of the authors and do not necessarily represent those of their affiliated organizations, or those of the publisher, the editors and the reviewers. Any product that may be evaluated in this article, or claim that may be made by its manufacturer, is not guaranteed or endorsed by the publisher.
